# Discovering Ni/Cu Single-Atom Alloy as a Highly Active and Selective Catalyst for Direct Methane Conversion to Ethylene: A First-Principles Kinetic Study

**DOI:** 10.1021/acscatal.5c02570

**Published:** 2025-06-20

**Authors:** Manish Kothakonda, Sarah LaCroix, Chengyu Zhou, Ji Yang, Ji Su, Qing Zhao

**Affiliations:** † Department of Chemical Engineering, 1848 Northeastern University, Boston, Massachusetts 02115, United States; ‡ Energy Storage and Distributed Resources Division, 1666 Lawrence Berkeley National Laboratory, Berkeley, California 94720, United States

**Keywords:** single-atom alloy, methane activation, first-principles simulation, thermal catalysis, methane to ethylene conversion

## Abstract

Direct methane conversion to liquid fuels or value-added chemicals is a promising technology to utilize natural resources without resorting to further petroleum extraction. However, discovering efficient catalysts for this reaction is challenging due to either coke formation or unfavorable C–H bond activation. Herein, we design single-atom alloy (SAA) catalysts to simultaneously eliminate the above two bottlenecks based on mechanism-guided strategies: (1) the active single atom enables favorable C–H bond breaking and (2) the less reactive host metal facilitates C–C coupling and thus avoids strong binding of carbonaceous species. Employing electronic structure theory calculations, we screened the stability of multiple SAAs with 3d-5d transition metals atomically dispersed on a copper surface in terms of avoiding dopant aggregation and segregation. We then evaluated reactivities of the stable SAAs as catalysts for direct methane conversion to C_2_ products, including methane dehydrogenation and C–C coupling mechanisms. Combining selectivity analysis with kinetic modeling, we predicted that nickel dispersed on copper, i.e., Ni/Cu SAA, is a highly active and selective catalyst that can efficiently transform methane to ethylene. This work designs efficient SAA catalysts for direct methane activation and provides chemical insights into engineering compositions of SAAs to tune their catalytic performances.

## Graphical abstract

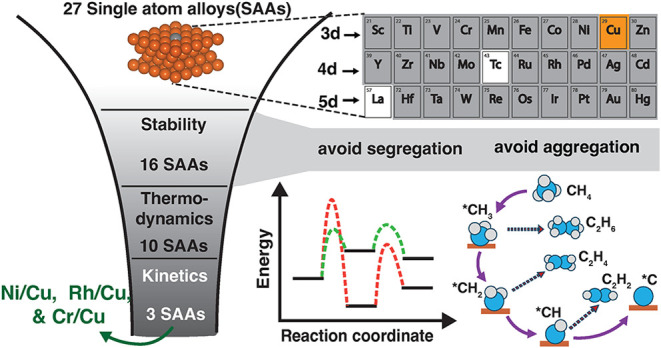

## I Introduction

In the foreseeable future, carbon-based fuels will continue to dominate our energy consumption. Development of robust catalytic materials to meet our daily energy needs is arguably the most critical scientific and engineering challenge.^1–7^ Methane (CH_4_), as the primary component of natural gas, methane clathrates,^8,9^ and shale gas,^10^ is poised to become the most crucial hydrocarbon feedstock for fuel and chemical synthesis, providing an economical alternative to the declining petroleum reserves. Despite the vast availability of methane, it requires a complex infrastructure for secure transportation, storage, and distribution, limiting efficient usage of natural resources. In addition, methane is the second most abundant greenhouse gas with potential to contribute to global warming that is approximately 25 times greater than carbon dioxide (CO_2_) over the next one hundred years.^11^ Therefore, there is an urgent need to discover low-carbon footprint methods to selectively convert methane to transportable, high-energy-density liquid fuels and value-added chemical feedstocks.

Although methane conversion pathways are desirable, the technologies are not yet competitive with petroleum-based production of chemicals and fuels. Industrially, methane conversion could be realized in large scale through steam methane reforming to produce syngas,^12^ a mixture of carbon monoxide (CO) and hydrogen gas (H_2_), which are then used as feedstocks in the Fischer–Tropsch synthesis process^13,14^ to generate liquid hydrocarbons. However, this process is energy-intensive and generates carbonaceous deposits (coke), leading to catalyst deactivation.^15,16^ In addition, the subsequent Fischer–Tropsch process requires either CO or H_2_ to remove oxygen from CO, reducing the carbon atom utilization efficiency or consuming valuable H_2_ resources. Thus, direct methane conversion to multicarbon hydrocarbons operating under mild conditions without going through the intermediate syngas production is more economically and environmentally desirable. A variety of heterogeneous catalysts have been explored as catalytic materials for direct methane conversion, including oxidative coupling of methane over metal oxides,^17,18^ non-oxidative methane dehydroaromatization using zeolite-supported metal catalysts,^19,20^ and direct conversion through C–C coupling on metallic surfaces.^21^ Unfortunately, no practical catalysts exist today for direct methane conversion due to high C–H bond activation barriers and/or rapid catalyst deactivation by coke contamination.^22–24^

Recently, Marcinkowski et al.^25^ and Hannagan et al.^26^ experimentally demonstrated that single-atom alloys (SAAs), atomically dispersing a minimum amount of reactive metal atoms in the surface layer of a less reactive host metal, can be used as efficient and coke-resistant catalysts for dehydrogenation of alkanes to produce alkenes. More specifically, platinum (Pt) dispersed in the surface layer of copper (Cu), i.e., Pt/Cu SAA (the notation A/B denotes the single atom A dispersed in the surface layer of host B), successfully activates desirable C–H bonds but exhibits high barriers for subsequent dehydrogenation steps that lead to coke formation,^25^ outperforming its pure metal components in that Pt can activate C–H bonds but suffers from coking,^16,23^ while Cu is resistant to coke formation but exhibits a high C–H activation barrier.^21^ Similarly, rhodium (Rh) dispersed in the surface layer of Cu, i.e., Rh/Cu SAA, was designed as a catalyst for propane dehydrogenation to propene, while either Rh or Cu pure metal is not generally considered as a catalyst for alkane dehydrogenation.^26^ The primary advantage of SAAs lies in their unique geometries to decouple the location of the transition state and binding sites of the reaction intermediates, allowing simultaneously favorable bond dissociations and weak binding energies and thus enhance catalyst activity and selectivity.^27^

Moreover, the well-defined active sites in SAAs make the understanding of reaction mechanisms and thus prediction of reactivity relatively easy with density functional theory (DFT), which is the most popular computational modeling tool for heterogeneous catalysis,^28–33^ to accelerate the discovery of catalytically efficient SAAs. Motivated by the success of SAAs for alkane dehydrogenation, we began to use DFT to design SAAs for converting methane to multicarbon hydrocarbons through engineering (1) single catalytic sites to enable facile C–H activation and (2) host metals to allow favorable C–C coupling and weak bindings of carbonaceous deposits. Specifically, we considered various 3d-5d transition metal atoms dispersed in the surface layer of Cu(111) surface as catalysts for direct methane conversion to C_2_ products, including ethane (C_2_H_6_), ethylene (C_2_H_4_), and acetylene (C_2_H_2_). Cu was selected as the host metal due to its kinetically favorable C–C coupling and weak bindings to adsorbates.^34,35^ The Cu(111) facet was chosen as it is the most stable Cu facet, and many Cu(111)-based SAAs have been successfully synthesized in experiments.^27^ Herein, we started by studying the stabilities of these Cu-based SAAs in terms of avoiding dopant aggregation and segregation. On stable SAAs, we then evaluated thermodynamics and performed detailed kinetic modeling of methane dehydrogenation to atomic carbon and multiple C–C coupling mechanisms between carbonaceous species, toward the ultimate goal of identifying active, selective, and coke-resistant SAAs for methane utilization.

## II Methods

We performed spin-polarized periodic DFT calculations with the all-electron, frozen-core, projector augmented-wave (PAW)^36^ method using the Vienna Ab initio Simulation Package (VASP), version 6.3.1.^37–40^ We used the Perdew–Burke–Ernzerhof (PBE)^41^ exchange-correlation functional together with Grimme’s D3 ^42,43^ dispersion correction and the Becke–Johnson damping function.^44^ We applied a kinetic-energy cutoff value of 500 eV for the plane-wave basis set. We utilized a 4 × 4 supercell containing four atomic layers (64 atoms) to simulate surfaces when evaluating the stability of SAAs and determining reaction pathways of direct methane conversion. We constructed a periodic cell with a vacuum spacing of at least 15 Å along the direction normal to the SAA surface, resulting in a unit cell length of 30 Å along the *c*-axis to prevent interactions between the periodic images. The atoms in the two topmost layers of the 4 × 4 four-layer slab, as well as all adsorbed species, were relaxed, while the atoms in the two bottommost layers were fixed at their bulk atomic positions to simulate the semi-infinite Cu bulk crystal structure. Γ-point-centered Monkhorst–Pack^45^ *k*-point grids of 15 × 15 × 15 and 5 × 5 × 1 were applied to sample the Brillouin zone for the bulk unit cell and the 4 × 4 periodic slabs, respectively. Benchmark calculations indicated that the selection of kinetic-energy cutoff (500 eV vs 1000 eV) and *k*-point grids (15 × 15 × 15 vs 24 × 24 × 24 for bulk and 5 × 5 × 1 vs 6 × 6 × 1 for slab) converged the total energies to 1 meV/atom. To aid the self-consistent field convergence, we used Methfessel-Paxton smearing^46^ for Brillouin zone integration with a smearing width of 0.1 eV. In geometry optimizations, forces were converged to 0.03 eV/Å. We applied dipole-field energy and potential corrections^47^ to cancel the artificial field interaction between the slabs along the direction normal to the SAA surface.

We considered the aggregation energy and segregation energy when predicting the stability of SAAs. Aggregation energy quantifies the relative stability of the single isolated dopant atom toward aggregation, i.e., forming dopant clusters, on the host metal, and was computed using^27,48,49^

$$\Delta E_{\mathrm{agg}}\left(n\right)=E_{\mathrm{tot}}\left(n\right)+\left(n-1\right)E_{\mathrm{tot}}\left(\mathrm{host}\right)-nE_{\mathrm{tot}}\left(\mathrm{SAA}\right)$$

in which *E*_tot_(*n*), *E*_tot_(host), and *E*_tot_(SAA) are the total energies of an alloy surface with a cluster of *n* dopant atoms, a pure host surface, and an SAA surface, respectively. Here, we used a dopant dimer embedded on the alloy surface to represent the formation of dopant clusters in the prediction of aggregation energy, leading to *n* = 2 in the above equation. Segregation energy indicates the relative stability of the single isolated dopant atom in the surface layer of the host, i.e., forming an SAA, versus immersed in the bulk host materials, and was calculated using^27,48,49^

$$\Delta E_{\mathrm{seg}}=E_{\mathrm{tot}}\left(\mathrm{bulk}\right)-E_{\mathrm{tot}}\left(\mathrm{SAA}\right)$$

in which *E*_tot_(bulk) and *E*_tot_(SAA) are the total energies of an alloy surface with a dopant immersed in the bulk host slab and an SAA surface, respectively. For each dopant/host SAA combination, four different slab models, either alloys or pure metal, are essential to compute the aggregation and segregation energies (see the Supporting Information Figure S1 for representative slab models). With the above formulations, a positive aggregation energy and a positive segregation energy indicate the formation of an SAA. We optimized the minimum energy paths (MEPs) for CH_4_ dehydrogenation to *C (adsorbed carbon; * refers to an adsorption site) and C–C coupling mechanisms to ethane and ethylene using the climbing image nudged elastic band (CI-NEB) method^50^ with an artificial spring force constant of 3 eV/Å^2^ along the reaction tangents.

## III Results and Discussion

### III.I Evaluating the Structural and Energetic Stability of SAAs

In this study, we investigated 27 SAAs consisting of 3d-5d transition metals dispersed in the surface layer of a Cu(111) surface. Two dopants, lanthanum (La) and technetium (Tc), were excluded from this study since it is hard to obtain a nicely converged geometry with a reliable electronic structure for the La doped on Cu (La/Cu) SAA, as well as the radioactive nature of Tc for experimental validation and safe handling of the Tc doped on Cu (Tc/Cu) SAA. When predicting the structural and energetic stability of an SAA, we considered both aggregation and segregation energies. Aggregation energy quantifies the thermodynamic stability of forming isolated dopants versus forming dopant clusters on the host metal, while segregation energy computes the relative stability of the isolated dopant staying in the surface layer or segregate into the bulk of the host.^27,48,49^ A dopant/host combination with both positive aggregation and segregation energies maintains an isolated atomic configuration of the dopant, forming an SAA, while preventing the formation of clusters, immersed dopants, or intermetallic phases (*vide supra*).

DFT calculations often neglect entropic contributions that typically favor dispersion of the dopants, and thus dopant/host combinations with aggregation and/or segregation energies that are negative but close to zero may still be accessible in experimental synthesis operated at elevated temperatures. Assuming a 0.25 eV entropic contribution, we identified 16 stable SAAs out of the 27 candidates studied, including 3d dopants of Sc/Cu, Cr/Cu, Mn/Cu, Ni/Cu, and Zn/Cu; 4d dopants of Y/Cu, Zr/Cu, Rh/Cu, Pd/Cu, Ag/Cu, and Cd/Cu; 5d dopants of Hf/Cu, Ir/Cu, Pt/Cu, Au/Cu, and Hg/Cu (Figure 1, SI Figure S2, and Table S1). Unsurprisingly, several DFT-predicted stable SAAs have been successfully synthesized in recent experiments, demonstrating the robustness of our computational strategy to predict the stability. For example, the Ni/Cu SAA was prepared by a modified electroless galvanic deposition method and was characterized using X-ray powder diffraction (XRD), high-resolution transmission electron microscopy (HRTEM), and scanning electron microscopy (SEM) images for ethanol dehydrogenation.^51,52^ In another work, the Rh/Cu SAA was synthesized and examined using multiple experimental techniques to understand the active single site, including low-temperature scanning tunneling microscopy (LS-STM), temperature programed desorption (TPD), and reflection absorption infrared spectroscopy (RAIRS).^53^ Using similar synthesis and characterization techniques, Pd/Cu SAA^54,55^ and Pt/Cu SAA^56^ have also been successfully prepared. In addition to experimental verification, our DFT-predicted stability of SAAs is consistent with other simulation studies.^48,49,57^

**Figure 1:**
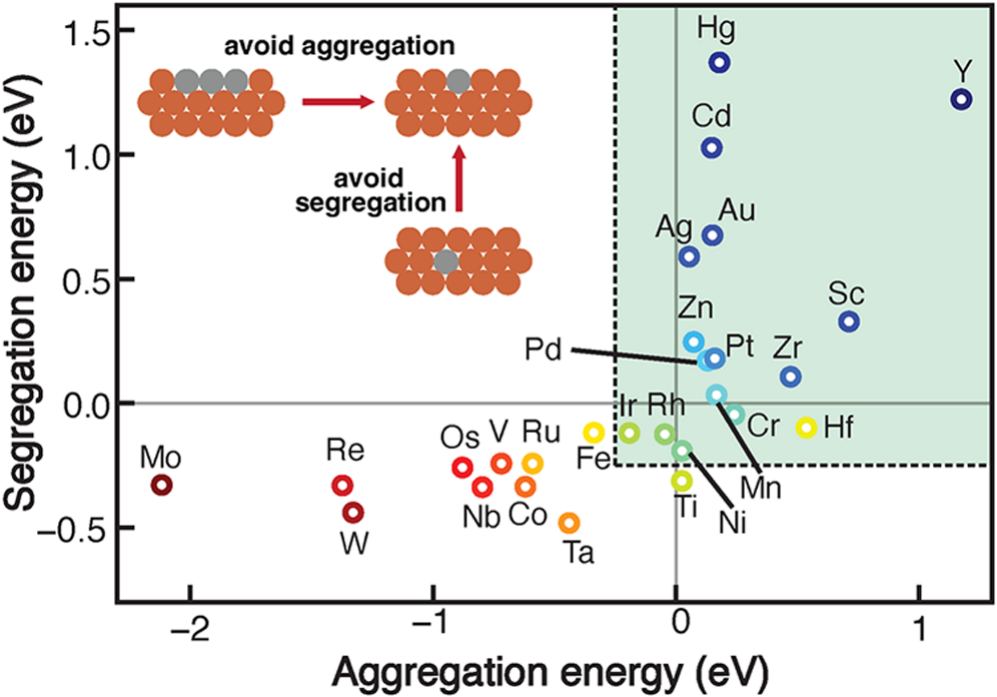
Segregation energy plotted against aggregation energy for 27 SAAs with 3d-5d transition metals dispersed in the surface layer of Cu(111). The green shaded region indicates the formation of an SAA with both positive aggregation energy and positive segregation energy assuming a 0.25 eV entropic contribution. SAAs are labeled using the dopant element. Dark blue circles indicate the most stable SAAs, while dark red circles indicate the least stable SAAs. Schematic of avoiding forming a dopant cluster (i.e., aggregation) or dopant immersing in the bulk (i.e., segregation) of the host metal is shown in the inset.

### III.II Direct Methane Conversion to C_2_ Hydrocarbons (Ethane, Ethylene, and Acetylene) on Stable SAAs

To understand the activity and selectivity of using SAAs for direct methane conversion to C_2_ products (ethane, ethylene, and acetylene), we explored reaction mechanisms of full dehydrogenation of methane to adsorbed carbon (*C) and various C–C coupling mechanisms of partially dehydrogenated species, including methyl (*CH_3_) coupling to ethane, methylene (*CH_2_) coupling to ethylene, and methyne (*CH) coupling to acetylene (Figure 2a), on identified stable SAAs (*vide supra*). We first screened the most favorable adsorption sites for all methane dehydrogenation intermediates (*CH_3_, *CH_2_, *CH, *C, and *H) on stable SAAs (SI Figures S3 and S4, and Table S2). Here, we only focused on the adsorption sites around dopants since experimental work characterized them as the active sites.^25^ For most SAAs, intermediates tend to consistently favor the hollow site, with exceptions showing preferences for the top or bridge site for *CH_3_ and *CH_2_ intermediates. These differences suggest that the choice of the dispersed dopant can influence the preferred adsorption sites of intermediates, potentially affecting the reactivity of the catalysts.

**Figure 2:**
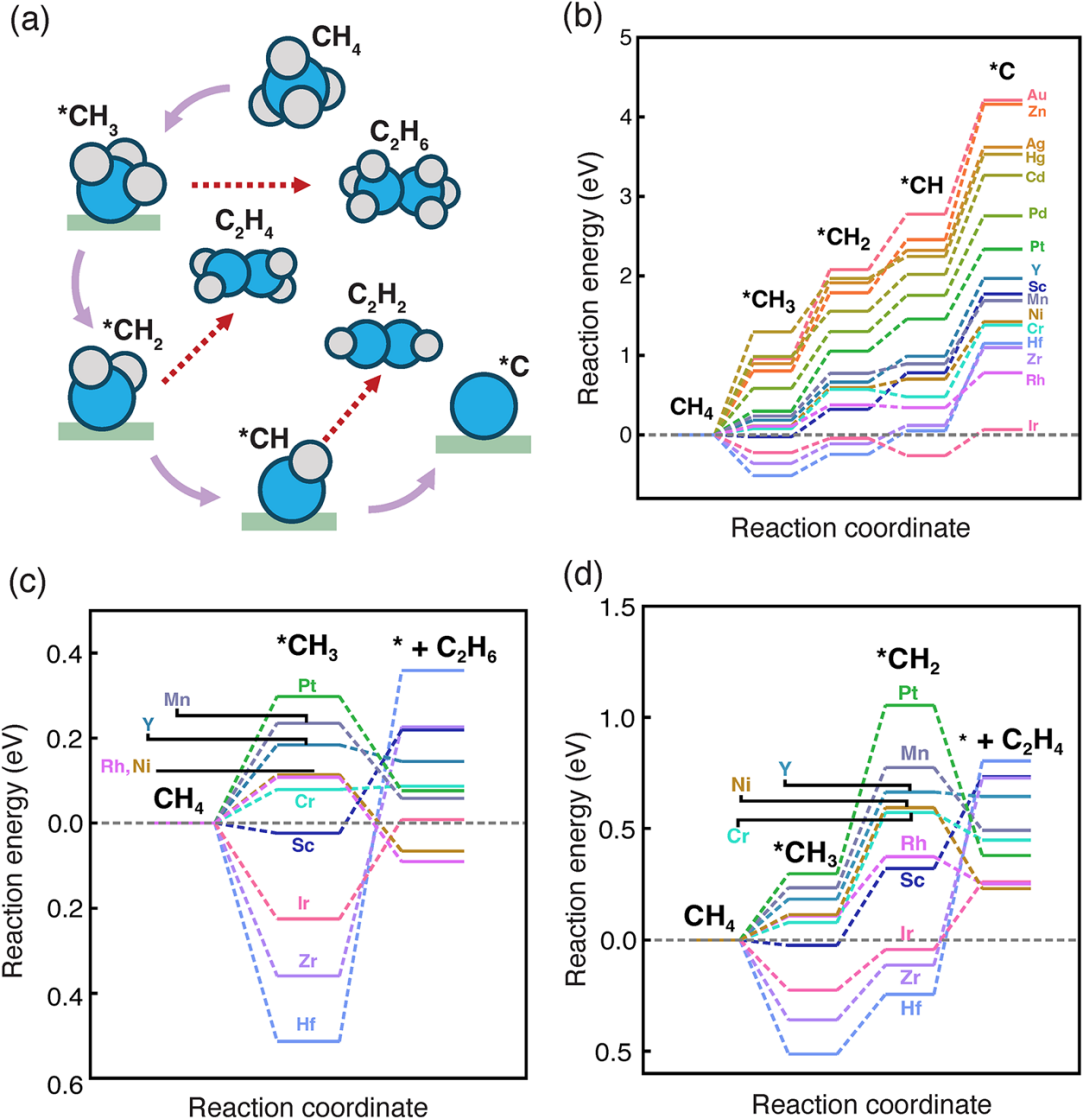
(a) Schematic of methane dehydrogenation mechanisms to *C and C–C coupling steps leading to ethane, ethylene, and acetylene formation. Atoms are colored as follows: blue for carbon and gray for hydrogen. Solid arrows indicate methane dehydrogenation, and dashed arrows indicate C–C coupling. (b) Full-energy landscapes of methane dehydrogenation to *C on 16 stable SAAs. Full-energy landscapes of direct methane activation to (c) ethane and (d) ethylene on 10 stable SAAs with a reaction energy less than 0.5 eV for the first methane dehydrogenation step. SAAs are labeled using the dopant element. For C–C coupling steps shown in (c) and (d), the energies are for a whole C–C coupling step, i.e., *CH_3_ + *CH_3_ → C_2_H_6_ in (c) and *CH_2_ + *CH_2_ → C_2_H_4_ in (d).

Starting from the most preferred adsorption sites, we first calculated reaction energies of CH_4_ dehydrogenation to *C on 16 stable SAAs (Figure 2b, and SI Table S3). We observed that dopants can effectively tune the activity of C–H activation on the Cu(111) host, resulting in a wide range of dehydrogenation energies: −0.51 eV (Hf/Cu) to 1.30 eV (Hg/Cu) for CH_4_ dehydrogenation; 0.18 eV (Ir/Cu) to 1.12 eV (Au/Cu) for *CH_3_ dehydrogenation; −0.22 eV (Ir/Cu) to 0.70 eV (Au/Cu) for *CH_2_ dehydrogenation; 0.33 eV (Ir/Cu) to 1.70 eV (Zn/Cu) for *CH dehydrogenation (SI Table S3). The last dehydrogenation step, i.e., *CH to *C, is usually the least favorable step with the highest reaction energies. In addition, we noted a correlation between the C–H activation energies for the first two dehydrogenation steps (i.e., CH_4_ to *CH_3_ and *CH_3_ to *CH_2_) and the dopant’s outer-shell *d*-electron configuration that reaction energies become more positive with increasing number of outer-shell *d*-electrons, while the other two dehydrogenation steps (i.e., *CH_2_ to *CH and *CH to *C) exhibit different trends that reaction energies become more negative first and then more positive with increasing number of *d*-electrons of dopants (SI Figure S5). In general, SAAs with dopants from early and midrow transition metals show surmountable reaction energies for CH_4_ dehydrogenation, while SAAs containing late transition metals from groups 11 and 12 consistently exhibit higher CH_4_ dehydrogenation energies. This trend suggests that SAAs containing dopants with almost filled *d* orbitals are not reactive to C–H bond activation. We analyzed the dopant *d*-band center of SAAs and noted mixed behaviors across different intermediate-adsorbed SAA surfaces (SI Figure S6). In addition, the most preferred adsorption site is inconsistent for different carbonaceous species (SI Table S2). Both phenomena contribute to the divergent correlation between the d-electrons of dopants and C–H activation steps.

We next investigated possible C–C coupling steps toward producing C_2_ hydrocarbons, including ethane, ethylene, and acetylene. We considered coupling between two *CH_3_ intermediates to ethane, two *CH_2_ intermediates to ethylene, and two *CH intermediates to acetylene on 16 stable SAAs (Figure 2, and SI Table S3). Similar to CH_4_ dehydrogenation steps, dopants in SAAs affect the activity of C–C coupling steps, leading to wide ranges of reaction energies: −1.95 eV (Hg/Cu) to 0.87 eV (Hf/Cu) for ethane formation; −1.96 eV (Hg/Cu) to 1.05 eV (Hf/Cu) for ethylene generation; −0.94 eV (Au/Cu) to 1.76 eV (Zr/Cu) for acetylene production (SI Table S3). Interestingly, we observed divergent trends from dehydrogenation steps that SAAs with dopants from late transition metals are more reactive for C–C coupling. That is to say, the outer-shell *d*-electron configuration of dopants in SAAs also serves as a descriptor for C–C coupling reactivity. Indeed, we found a strong correlation that reaction energies of all three C–C coupling steps become more negative with an increasing number of outer-shell *d*-electrons of dopants (SI Figure S7).

### III.III. Selectivity Analysis

Overall, Cu(111)-based SAAs exhibit an inverse correlation between CH_4_ dehydrogenation and C–C coupling steps (SI Figure S8); i.e., reactive SAAs for C–H bond activation are inert for C–C coupling and vice versa. Therefore, SAAs with dopants from midrow transition metals that balance well two types of chemistries are potential active and selective catalysts for direct methane conversion to multicarbon hydrocarbons. To quantitatively analyze SAA activity and selectivity, we compared both the first CH_4_ dehydrogenation energy (i.e., CH_4_ to *CH_3_) and an energy difference, Δ*E*, between reaction energies of the second dehydrogenation step (i.e., *CH_3_ to *CH_2_) and the two *CH_3_ coupling reaction (Figure 3). A low CH_4_ dehydrogenation energy indicates that the catalyst is active for initial C–H bond breaking, while a high Δ*E* means that the catalyst is selective for C–C coupling toward ethane formation while suppressing further dehydrogenation. We selected the Δ*E* between these two particular steps because thermodynamics tells us that reaction energies of *CH_3_ dehydrogenation to *CH_2_ are positive on all SAA catalysts (Figure 2b), while reaction energies of two *CH_3_ coupling to ethane could be negative on some active SAA catalysts (Figure 2c), indicating that ethane is likely to be the dominant product for direct methane conversion on SAA catalysts. Following this analysis, we defined an SAA that simultaneously has less than 0.15 eV for CH_4_ dehydrogenation and greater than 0.45 eV for Δ*E* as an active and selective catalyst for direct methane conversion and found that Ni/Cu, Cr/Cu, and Rh/Cu SAAs fall in this region that can potentially transform methane to multicarbon hydrocarbons (Figure 3). The selection of 0.15 eV reaction energy for CH_4_ dehydrogenation activity and 0.45 eV energy difference for C–C coupling selectivity is based on a recent work^25^ of identifying Pt/Cu SAA for efficient C–H activation. Based on their DFT study,^25^ pure Cu(111) and Pt(111) have reaction energies of 0.61 and −0.27 eV for the first dehydrogenation step of CH_4_, respectively. A practical catalyst should have a reaction energy within the range of −0.27 to 0.61 eV that can activate the C–H bond but avoid the problem of coke formation. Therefore, we selected a C–H activation energy of 0.15 eV, which is in the middle of the range to represent a cutoff criterion. While for the selectivity of C–C coupling vs C–H activation, we observed a linear scaling relationship between the reaction energy and reaction barrier of all C–H bond activation steps on pure Cu(111), pure Pt(111), and Pt/Cu SAA reported in their work.^25^ Based on the slope, a reaction energy difference of 0.45 eV corresponds to a reaction barrier difference of 1.10 eV, which is large enough to enhance the selectivity toward C–C coupling. We highlight that the selection of these criteria for kinetic study is somewhat arbitrary since the scope of our work is to establish a systematic workflow of identifying SAA catalysts for direct methane conversion from evaluating stability and thermodynamics to kinetics. Development of catalyst design principles of SAA catalysts for direct methane conversion necessitates a kinetic study of a broader SAA chemical space, which is subject to ongoing work in the group.

**Figure 3:**
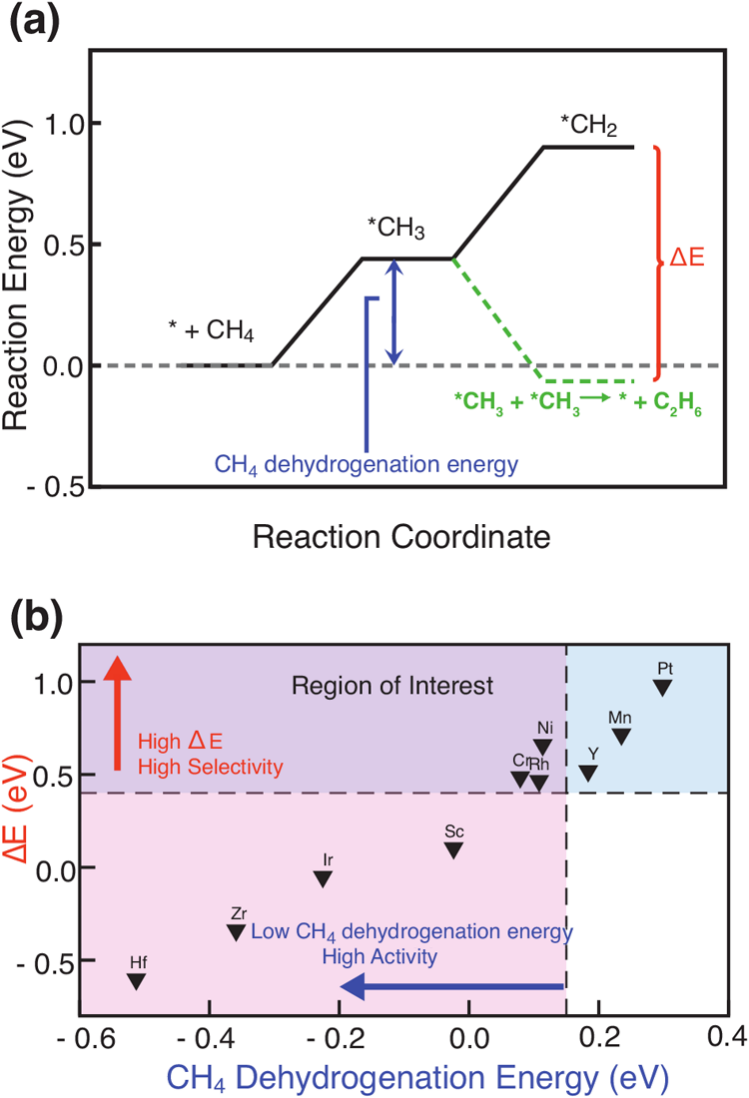
(a) Schematic of the energy landscape showing the reaction energy of the first methane dehydrogenation step and the energy difference (Δ*E*) between *CH_3_ dehydrogenation and two *CH_3_ coupling to ethane. For the C–C coupling step, the energy is for a whole C–C coupling step, i.e., *CH_3_ + *CH_3_ → C_2_H_6_. (b) Δ*E* plotted against CH_4_ dehydrogenation energy for 10 stable SAAs with methane dehydrogenation energy less than 0.5 eV. The vertical black dashed line indicates a methane dehydrogenation energy of 0.15 eV with pink and purple shaded regions on the left representing SAAs with high activity for methane activation. The horizontal black dashed line indicates a Δ*E* of 0.40 eV with blue and purple shaded regions above representing SAAs with high selectivity for ethane production. SAAs in the purple shaded region (i.e., Ni/Cu, Cr/Cu, and Rh/Cu) are active and selective catalysts for direct methane conversion to multicarbon hydrocarbons.

### III.IV. Kinetic Modeling of Direct Methane Conversion to C_2_ Products on Ni/Cu, Rh/Cu, and Cr/Cu SAAs

The selection of Ni/Cu, Rh/Cu, and Cr/Cu SAA catalysts for direct methane conversion to multicarbon hydrocarbons is guided by thermodynamics of CH_4_ dehydrogenation and C–C coupling mechanisms. To fully understand their reactivities, evaluation of the kinetics, i.e., predicting reaction barriers, is essential. We started from optimizing the MEP on the Ni/Cu SAA (Figure 4a). The initial dehydrogenation step of CH_4_ to *CH_3_ starts from a desorbed CH_4_ molecule on top of the SAA surface and then undergoes C–H bond breaking close to the Ni dopant site in the transition state toward forming an adsorbed *CH_3_ at an atop site and a *H at a hollow site. This C–H activation step is both thermodynamically and kinetically favorable, with a reaction energy of 0.11 eV and a barrier of 0.77 eV (Figure 4b), indicating that this step can proceed under mild conditions. The second dehydrogenation step (*CH_3_ to *CH_2_) proceeds via a transition state of C–H bond breaking close to the Ni dopant site toward both products (i.e., *CH_2_ and *H) moving to hollow sites with a reaction energy of 0.48 eV and a barrier of 0.75 eV (Figure 4b). The competing C–C coupling between two *CH_3_ intermediates to form ethane is thermodynamically more favorable with a reaction energy of −0.18 eV but kinetically less favorable with a higher barrier of 1.07 eV, indicating that *CH_2_ is more likely to be the dominant intermediate though the products are dependent on the operating temperature. As the pathway progresses, dehydrogenation of *CH_2_ to *CH occurs with a reaction energy of 0.11 eV and a barrier of 0.50 eV. However, the C–C coupling of two *CH_2_ to form ethylene is extremely favorable as a barrierless process with a reaction energy of −0.36 eV, indicating a very fast reaction rate for ethylene formation. Previous study shows a *CH_2_ dimerization barrier of 0.46 eV on a pure Cu surface,^58^ suggesting the synergy between the Ni dopant and the Cu host in promoting C–C coupling. Since *CH_2_ to *CH is both thermodynamically and kinetically hindered, we did not consider the kinetics of further dehydrogenation (i.e., *CH to *C) and two *CH coupling. In addition, we evaluated the kinetics of adsorbed hydrogen associative desorption from the Ni/Cu SAA surface, i.e., *H + *H → H_2_, to understand whether the active sites can be blocked by hydride formation from the first and second dehydrogenation steps. The predicted reaction barrier is 0.80 eV, including that hydride is unlikely on the surface and thus the surface remains catalytically active rather than being contaminated by adsorbed hydrogen. Overall, we identified Ni/Cu as a highly active and selective catalyst for direct methane conversion to ethylene with the rate-limiting step being CH_4_ dehydrogenation to *CH_3_ and a barrier of 0.77 eV. To the best of our knowledge, no theoretical prediction and experimental investigation exist for using the Ni/Cu SAA catalyst for direct methane transformation to ethylene. To experimentally verify the theory-guided catalyst discovery, we are currently collaborating with experimentalists to understand the effects of temperature and dopant concentration on tuning the activity and selectivity of Ni/Cu SAA for this chemistry.

**Figure 4:**
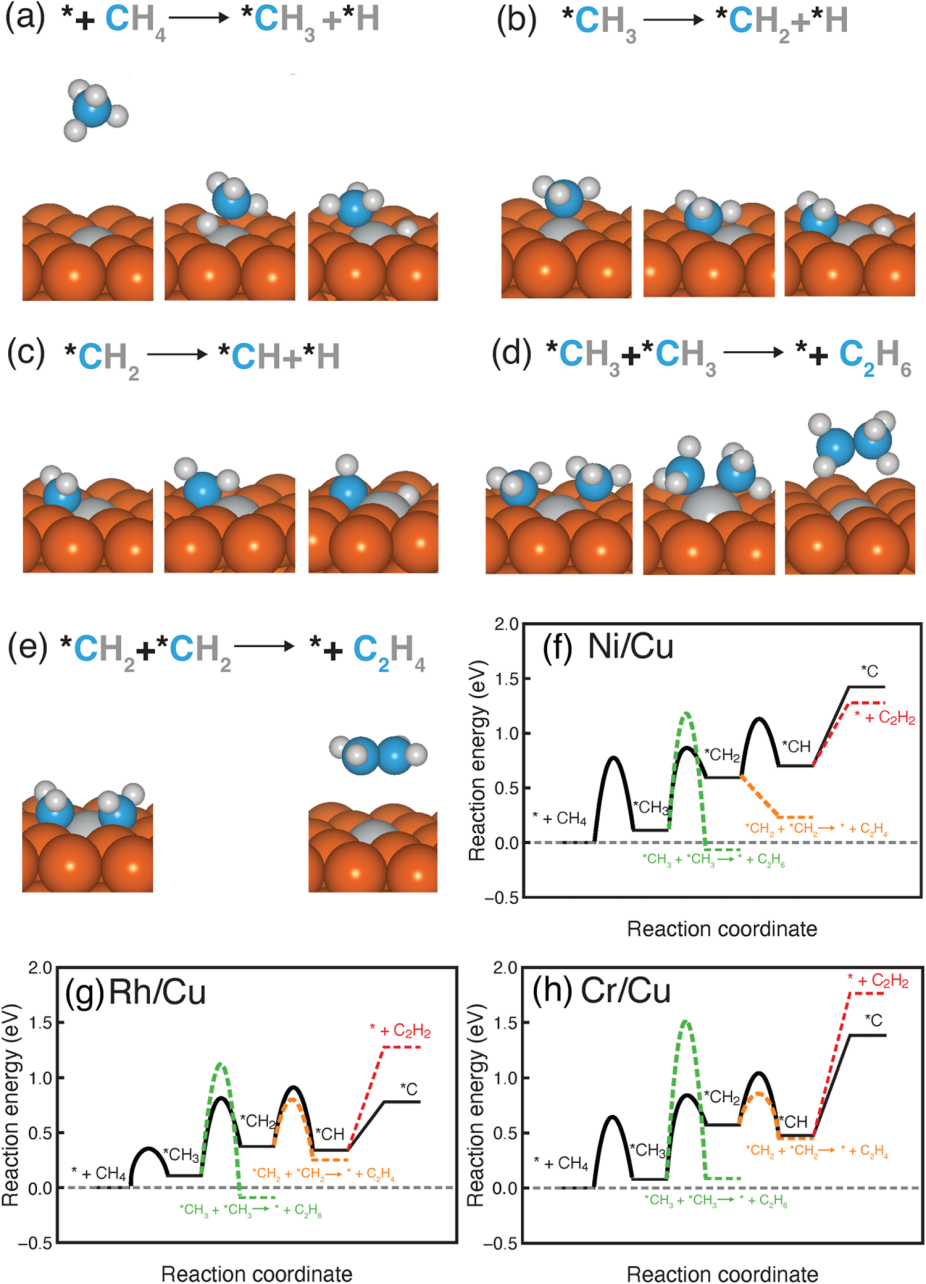
Critical structures (initial state, transition state, and final state) for (a–c) methane dehydrogenation and (d, e) C–C coupling mechanisms leading to C_2_ products on the Ni/Cu SAA. Energy landscapes for the full methane dehydrogenation to *C and C–C coupling steps, leading to the formation of ethane, ethylene, and acetylene on (f) Ni/Cu SAA, (g) Rh/Cu SAA, and (h) Cr/Cu SAA. Black solid lines indicate methane dehydrogenation. Dashed lines represent C–C coupling mechanisms (green for two *CH_3_ coupling to ethane, orange for two *CH_2_ coupling to ethylene, and red for two *CH coupling to acetylene). Reaction barriers for *CH dehydrogenation to *C and two *CH couplings to acetylene are not included in the energy landscapes. For C–C coupling steps shown in (f–h), the energies are for a whole C–C coupling step, i.e., *CH_3_ + *CH_3_ → C_2_H_6_ and *CH_2_ + *CH_2_ → C_2_H_4_.

We next considered the Rh/Cu SAA (Figure 4c and SI Figure S9). The first dehydrogenation step (CH_4_ to *CH_3_) is also energetically favorable with a reaction energy of 0.11 eV and a very low barrier of 0.35 eV (Figure 4c). The second dehydrogenation step (*CH_3_ to *CH_2_) proceeds with a reaction energy of 0.27 eV and a barrier of 0.71 eV. The competing C–C coupling of two *CH_3_ intermediates to ethane is thermodynamically more favorable with a reaction energy of −0.20 eV and a higher barrier of 1.01 eV. Similar to Ni/Cu, although thermodynamics favor ethane generation, kinetics prefers the formation of the *CH_2_ intermediate. Following that, further dehydrogenation of *CH_2_ to *CH has a reaction energy of −0.04 eV and a barrier of 0.53 eV. The other possibility is coupling between two *CH_2_ intermediates to form ethylene. Unlike the barrierless process of this step on Ni/Cu, Rh/Cu has a reaction energy of −0.13 eV and a barrier of 0.42 eV, indicating that the formation of ethylene is slightly favorable than the *CH intermediate and the coexistence of both species. Further dehydrogenation to *C and coupling between two *CH to acetylene are thermodynamically unfavorable, and thus kinetics is not considered here. Overall, the Rh/Cu SAA could be an active catalyst for ethylene production, although *CH intermediate might also exist and block the active sites.

We then studied the Cr/Cu SAA (Figure 4d and SI Figure S10). The first dehydrogenation step is energetically favorable, with a reaction energy of 0.08 eV and a barrier of 0.64 eV (Figure 4d). The second dehydrogenation step has a reaction energy of 0.48 eV and a barrier of 0.76 eV. Similar to the other two SAAs, *CH_3_ coupling is kinetically unfavorable with a sufficiently high barrier of 1.43 eV, indicating *CH_2_ as the only possible product. The subsequent dehydrogenation of *CH_2_ to *CH proceeds with a reaction energy of −0.09 eV and a barrier of 0.47 eV. However, C–C coupling of two *CH_2_ to ethylene is even more favorable with a similar reaction energy of −0.12 eV and a lower barrier of 0.28 eV, suggesting that ethylene is a product for methane conversion on the Cr/Cu SAA. Further reactions to either *C or acetylene are impossible with very high reaction energies. Therefore, Cr/Cu also emerges as an efficient catalyst for direct methane conversion to ethylene.

## IV Conclusions

To summarize, we explored the possibility of using Cu(111)-based SAAs with dopants from 3d-5d transition metals as catalysts for direct methane conversion to produce C_2_ products including ethane, ethylene, and acetylene. We started from assessing their stabilities by calculating the aggregation and segregation energies, ensuring that the dopant atoms remain singly dispersed on the surface of Cu(111). We identified 16 stable SAAs out of the 27 considered candidates. Several DFT-predicted stable SAAs have already been successfully synthesized in experimental studies, indicating the robustness of our computational methodologies in predicting SAA stability.

We next studied the reactivity of the 16 stable SAAs as catalysts for direct methane conversion to ethane, ethylene, and acetylene, including the mechanisms of CH_4_ dehydrogenation and three C–C coupling steps. We observed that the outer-shell *d*-electron configurations of dopants in SAAs play a critical role in determining both reactivities of CH_4_ dehydrogenation and C–C coupling steps but affect the two chemistries in a different way. Basically, SAAs with dopants from early and midrow transition metals favor C–H bond activation, while SAAs with dopants from late transition metals favor C–C coupling. By performing quantitative selectivity analysis, we identified that Ni/Cu SAA, Rh/Cu SAA, and Cr/Cu SAA exhibit high activity and selectivity toward generating multicarbon hydrocarbons based on thermodynamics favorability.

We further performed kinetic modeling on the three SAAs and concluded that the Ni/Cu SAA is a highly active and selective catalyst for direct methane conversion to produce ethylene, with the rate-limiting step being CH_4_ dehydrogenation to *CH_3_ and a barrier of 0.77 eV. Rh/Cu and Cr/Cu SAAs can also transform methane to generate ethylene but might suffer from a competitive reaction of *CH formation that can potentially block the active sites. This work designs a novel catalyst, atomically dispersing Ni atoms in the surface layer of Cu(111) (Ni/Cu SAA), for direct methane conversion to ethylene, and deepens our understanding of improving SAA activity and selectivity through engineering compositions and optimizing reaction conditions.

## Supplementary Material





## Notes

Aggregation and segregation energies of Cu(111)-based SAAs; favorable adsorption sites of intermediates; geometries of SAAs and critical structures along MEPs on Rh/Cu and Cr/Cu; energetics of CH_4_ dehydrogenation and C–C coupling steps; and correlation between energetics and dopant outer-shell *d*-electrons (PDF)
Compressed VASP structure files for the slab models (ZIP)

## References

[1] Nitopi S., Bertheussen E., Scott S. B., Liu X., Engstfeld A. K., Horch S., Seger B., Stephens I. E. L., Chan K., Hahn C., Nørskov J. K., Jaramillo T. F., Chorkendorff I. (2019). Progress and Perspectives of Electrochemical CO_2_ Reduction on Copper in Aqueous Electrolyte. Chem. Rev..

[2] Walter M. G., Warren E. L., McKone J. R., Boettcher S. W., Mi Q., Santori E. A., Lewis N. S. (2010). Solar Water
Splitting Cells. Chem. Rev..

[3] Montoya J. H., Seitz L. C., Chakthranont P., Vojvodic A., Jaramillo T. F., Nørskov J. K. (2017). Materials
for solar fuels and chemicals. Nat. Mater..

[4] Kulkarni A., Siahrostami S., Patel A., Nørskov J. K. (2018). Understanding
Catalytic Activity Trends in the Oxygen Reduction Reaction. Chem. Rev..

[5] Xu S., Carter E. A. (2019). Theoretical Insights
into Heterogeneous (Photo)­electrochemical
CO_2_ Reduction. Chem. Rev..

[6] Zhao Q., Martirez J. M. P., Carter E. A. (2021). Revisiting Understanding of Electrochemical
CO_2_ Reduction on Cu(111): Competing Proton-Coupled Electron
Transfer Reaction Mechanisms Revealed by Embedded Correlated Wavefunction
Theory. J. Am. Chem. Soc..

[7] Cai J., Zhao Q., Hsu W.-Y., Choi C., Liu Y., Martirez J. M. P., Chen C., Huang J., Carter E. A., Huang Y. (2023). Highly Selective Electrochemical
Reduction of CO_2_ into
Methane on Nanotwinned Cu. J. Am. Chem. Soc..

[8] Boswell R., Collett T. S. (2011). Current perspectives on gas hydrate resources. Energy Environ. Sci..

[9] Moore T. A. (2012). Coalbed
methane: a review. Int. J. Coal Geol..

[10] Wang Q., Chen X., Jha A. N., Rogers H. (2014). Natural gas from shale
formation–the evolution, evidences and challenges of shale
gas revolution in United States. Renewable Sustainable
Energy Rev..

[11] Agency U. S. E. P. . Understanding Global Warming Potentials. 2024.

[12] Barelli L., Bidini G., Gallorini F., Servili S. (2008). Hydrogen production
through sorption-enhanced steam methane reforming and membrane technology:
A review. Energy.

[13] York A. P., Xiao T., Green M. L. (2003). Brief Overview
of the Partial Oxidation
of Methane to Synthesis Gas. Top. Catal..

[14] Choudhary T. V., Choudhary V. R. (2008). Energy-Efficient
Syngas Production through Catalytic
Oxy-Methane Reforming Reactions. Angew. Chem.,
Int. Ed..

[15] Meloni E., Martino M., Palma V. (2020). A Short Review on Ni Based Catalysts
and Related Engineering Issues for Methane Steam Reforming. Catalysts.

[16] Swaan H., Kroll V., Martin G., Mirodatos C. (1994). Deactivation
of supported nickel catalysts during the reforming of methane by carbon
dioxide. Catal. Today.

[17] Gambo Y., Jalil A., Triwahyono S., Abdulrasheed A. (2018). Recent advances
and future prospect in catalysts for oxidative coupling of methane
to ethylene: A review. J. Ind. Eng. Chem..

[18] Ortiz-Bravo C. A., Chagas C. A., Toniolo F. S. (2021). Oxidative
coupling of methane (OCM):
An overview of the challenges and opportunities for developing new
technologies. J. Nat. Gas Sci. Eng..

[19] Xu Y., Lin L. (1999). Recent advances in
methane dehydro-aromatization over transition
metal ion-modified zeolite catalysts under non-oxidative conditions. Appl. Catal., A.

[20] Menon U., Rahman M., Khatib S. J. (2020). A critical
literature review of the
advances in methane dehydroaromatization over multifunctional metal-promoted
zeolite catalysts. Appl. Catal., A.

[21] Yang F., Koeller J., Ackermann L. (2016). Photoinduced
Copper-Catalyzed C–H
Arylation at Room Temperature. Angew. Chem.,
Int. Ed..

[22] Taccardi N., Grabau M., Debuschewitz J., Distaso M., Brandl M., Hock R., Maier F., Papp C., Erhard J., Neiss C. (2017). Gallium-rich Pd–Ga phases as supported liquid
metal catalysts. Nat. Chem..

[23] Jiang F., Zeng L., Li S., Liu G., Wang S., Gong J. (2015). Propane Dehydrogenation over Pt/TiO2–Al2O3 Catalysts. ACS Catal..

[24] Iglesias-Juez A., Beale A. M., Maaijen K., Weng T. C., Glatzel P., Weckhuysen B. M. (2010). A combined in situ time-resolved UV–Vis, Raman
and high-energy resolution X-ray absorption spectroscopy study on
the deactivation behavior of Pt and PtSn propane dehydrogenation catalysts
under industrial reaction conditions. J. Catal..

[25] Marcinkowski M. D., Darby M. T., Liu J., Wimble J. M., Lucci F. R., Lee S., Michaelides A., Flytzani-Stephanopoulos M., Stamatakis M., Sykes E. C. H. (2018). Pt/Cu single-atom alloys as coke-resistant catalysts
for efficient C–H activation. Nat. Chem..

[26] Hannagan R.
T., Giannakakis G., Réocreux R., Schumann J., Finzel J., Wang Y., Michaelides A., Deshlahra P., Christopher P., Flytzani-Stephanopoulos M. (2021). First-principles
design of a single-atom–alloy propane dehydrogenation catalyst. Science.

[27] Hannagan R. T., Giannakakis G., Flytzani-Stephanopoulos M., Sykes E. C. H. (2020). Single-Atom
Alloy Catalysis. Chem. Rev..

[28] Medford A. J., Vojvodic A., Hummelshøj J. S., Voss J., Abild-Pedersen F., Studt F., Bligaard T., Nilsson A., Nørskov J. K. (2015). From the
Sabatier principle to a predictive theory of transition-metal heterogeneous
catalysis. J. Catal..

[29] Chen B. W. J., Xu L., Mavrikakis M. (2021). Computational
Methods in Heterogeneous
Catalysis. Chem. Rev..

[30] Zhao Q., Kulik H. J. (2018). Where Does the Density
Localize in the Solid State?
Divergent Behavior for Hybrids and DFT+U. J.
Chem. Theory Comput..

[31] Zhao Q., Kulik H. J. (2019). Stable Surfaces That Bind Too Tightly:
Can Range-Separated
Hybrids or DFT+U Improve Paradoxical Descriptions of Surface Chemistry?. J. Phys. Chem. Lett..

[32] Zhao Q., Carter E. A. (2020). Revisiting Competing
Paths in Electrochemical CO_2_ Reduction on Copper via Embedded
Correlated Wavefunction
Theory. J. Chem. Theory Comput..

[33] Janet J. P., Zhao Q., Ioannidis E. I., Kulik H. J. (2017). Density functional
theory for modelling large molecular adsorbate–surface interactions:
a mini-review and worked example. Mol. Simul..

[34] Zhao Q., Martirez J. M. P., Carter E. A. (2022). Charting C–C
coupling pathways
in electrochemical CO_2_ reduction on Cu(111) using embedded
correlated wavefunction theory. Proc. Natl.
Acad. Sci. U.S.A..

[35] Zhao Q., Martirez J. M. P., Carter E. A. (2022). Electrochemical Hydrogenation of
CO on Cu(100): Insights from Accurate Multiconfigurational Wavefunction
MethodsClick to copy article link. J. Phys.
Chem. Lett..

[36] Blöchl P. E. (1994). Projector
augmented-wave method. Phys. Rev. B.

[37] Kresse G., Furthmüller J. (1996). Efficient
iterative schemes for ab initio total-energy
calculations using a plane-wave basis set. Phys.
Rev. B.

[38] Kresse G., Furthmüller J. (1996). Efficiency of ab-initio total energy
calculations for
metals and semiconductors using a plane-wave basis set. Comput. Mater. Sci..

[39] Kresse G., Hafner J. (1994). Ab initio molecular-dynamics
simulation of the liquid-metal–amorphous-semiconductor
transition in germanium. Phys. Rev. B.

[40] Kresse G., Hafner J. (1993). Ab initio molecular dynamics for liquid metals. Phys. Rev. B.

[41] Perdew J. P., Burke K., Ernzerhof M. (1996). Generalized Gradient Approximation
Made Simple. Phys. Rev. Lett..

[42] Grimme S., Antony J., Ehrlich S., Krieg H. (2010). A consistent and accurate
ab initio parametrization of density functional dispersion correction
(DFT-D) for the 94 elements H-Pu. J. Chem. Phys..

[43] Grimme S., Ehrlich S., Goerigk L. (2011). Effect of
the damping function in
dispersion corrected density functional theory. J. Comput. Chem..

[44] Becke A. D., Johnson E. R. (2005). A density-functional
model of the dispersion interaction. J. Chem.
Phys..

[45] Monkhorst H. J., Pack J. D. (1976). Special points for
Brillouin-zone integrations. Phys. Rev. B.

[46] Methfessel M., Paxton A. (1989). High-precision sampling for Brillouin-zone integration
in metals. Phys. Rev. B.

[47] Makov G., Payne M. C. (1995). Periodic boundary
conditions in ab initio calculations. Phys.
Rev. B.

[48] Darby M. T., Sykes E. C. H., Michaelides A., Stamatakis M. (2018). Carbon Monoxide
Poisoning Resistance and Structural Stability of Single Atom Alloys. Top. Catal..

[49] Fu Q., Luo Y. (2013). Catalytic Activity of Single Transition-Metal Atom
Doped in Cu(111)
Surface for Heterogeneous Hydrogenation. J.
Phys. Chem. C.

[50] Henkelman G., Uberuaga B. P., Jónsson H. (2000). A climbing
image nudged elastic band
method for finding saddle points and minimum energy paths. J. Chem. Phys..

[51] Shan J., Liu J., Li M., Lustig S., Lee S., Flytzani-Stephanopoulos M. (2018). NiCu single
atom alloys catalyze the C-H bond activation in the selective non-oxidative
ethanol dehydrogenation reaction. Appl. Catal.,
B.

[52] Patel D. A., Hannagan R. T., Kress P. L., Schilling A. C., Çınar V., Sykes E. C. H. (2019). Atomic-Scale
Surface Structure and
CO Tolerance of NiCu Single-Atom Alloys. J.
Phys. Chem. C.

[53] Hannagan R. T., Patel D. A., Cramer L. A., Schilling A. C., Ryan P. T., Larson A. M., Çınar V., Wang Y., Balema T. A., Sykes E. C. H. (2020). Combining STM,
RAIRS and TPD to Decipher the Dispersion and Interactions Between
Active Sites in RhCu Single-Atom Alloys. ChemCatChem.

[54] Baber A. E., Tierney H. L., Sykes E. C. H. (2010). Atomic-Scale Geometry and Electronic
Structure of Catalytically Important Pd/Au Alloys. ACS Nano.

[55] Marcinkowski M. D., Jewell A. D., Stamatakis M., Boucher M. B., Lewis E. A., Murphy C. J., Kyriakou G., Sykes E. C. H. (2013). Controlling a
spillover pathway with the molecular cork effect. Nat. Mater..

[56] Lucci F. R., Lawton T. J., Pronschinske A., Sykes E. C. H. (2014). Atomic Scale
Surface Structure of Pt/Cu(111) Surface Alloys. J. Phys. Chem. C.

[57] Rao K. K., Do Q. K., Pham K., Maiti D., Grabow L. C. (2020). Extendable
Machine Learning Model for the Stability of Single Atom Alloys. Top. Catal..

[58] Luo W., Nie X., Janik M. J., Asthagiri A. (2016). Facet Dependence
of CO_2_ Reduction Paths on Cu Electrodes. ACS Catal..

