# Supplementary material for: Discovering Ni/Cu Single-Atom Alloy as a Highly Active and Selective Catalyst for Direct Methane Conversion to Ethylene: A First-Principles Kinetic Study
Source: ACS Catal. 2025 Jun 20;15(13):11608–16. doi: 10.1021/acscatal.5c02570 (PMC12235591; doi:10.1021/acscatal.5c02570)
Supplement: Supplementary file 1 [file cs5c02570_si_001.pdf]

Supporting Information for

Discovering Ni/Cu Single Atom Alloy as a Highly Active and Selective Catalyst for Direct Methane Conversion to Ethylene: A First-Principles Kinetic Study

Manish Kothakonda,[1] Sarah LaCroix[1], Chengyu Zhou,[1] Ji Yang,[2] Ji Su,[2] and Qing Zhao[1*]

[1] Department of Chemical Engineering, Northeastern University, Boston, MA 02115, United States

[2] Energy Storage and Distributed Resources Division, Lawrence Berkeley National Laboratory, Berkeley, CA 94720, United States

***Corresponding author:** Qing Zhao (Email: q.zhao@northeastern.edu)

Contents

Figure S1. Representative slabs for aggregation and segregation energy calculations Page S2
Figure S2. Geometries of all stable single atom alloys Page S3
Figure S3. Adsorption sites of intermediates considered on SAAs Page S4
Figure S4. Favorable adsorption sites of all intermediates on Ni/Cu Page S5
Figure S5. Correlation between C-H activation energy and outer shell d electron Page S6
Figure S6. Projected density of states (PDOS) of dopant d orbitals Pages S7-8
Figure S7. Correlation between C-C coupling energy and outer shell d electron Page S9
Figure S8. Correlation between dehydrogenation and C-C coupling energies Page S10
Figure S9. Critical structures of CH_4 dehydrogenation to C_2 products on Rh/Cu Page S11
Figure S10. Critical structures of CH_4 dehydrogenation to C_2 products on Cr/Cu Page S12
Table S1. Aggregation and segregation energies of SAAs Page S13
Table S2. Favorable adsorption sites of CH_4 dehydrogenation intermediates Page S14
Table S3. Reaction energies of CH_4 dehydrogenation and C-C coupling steps Page S15

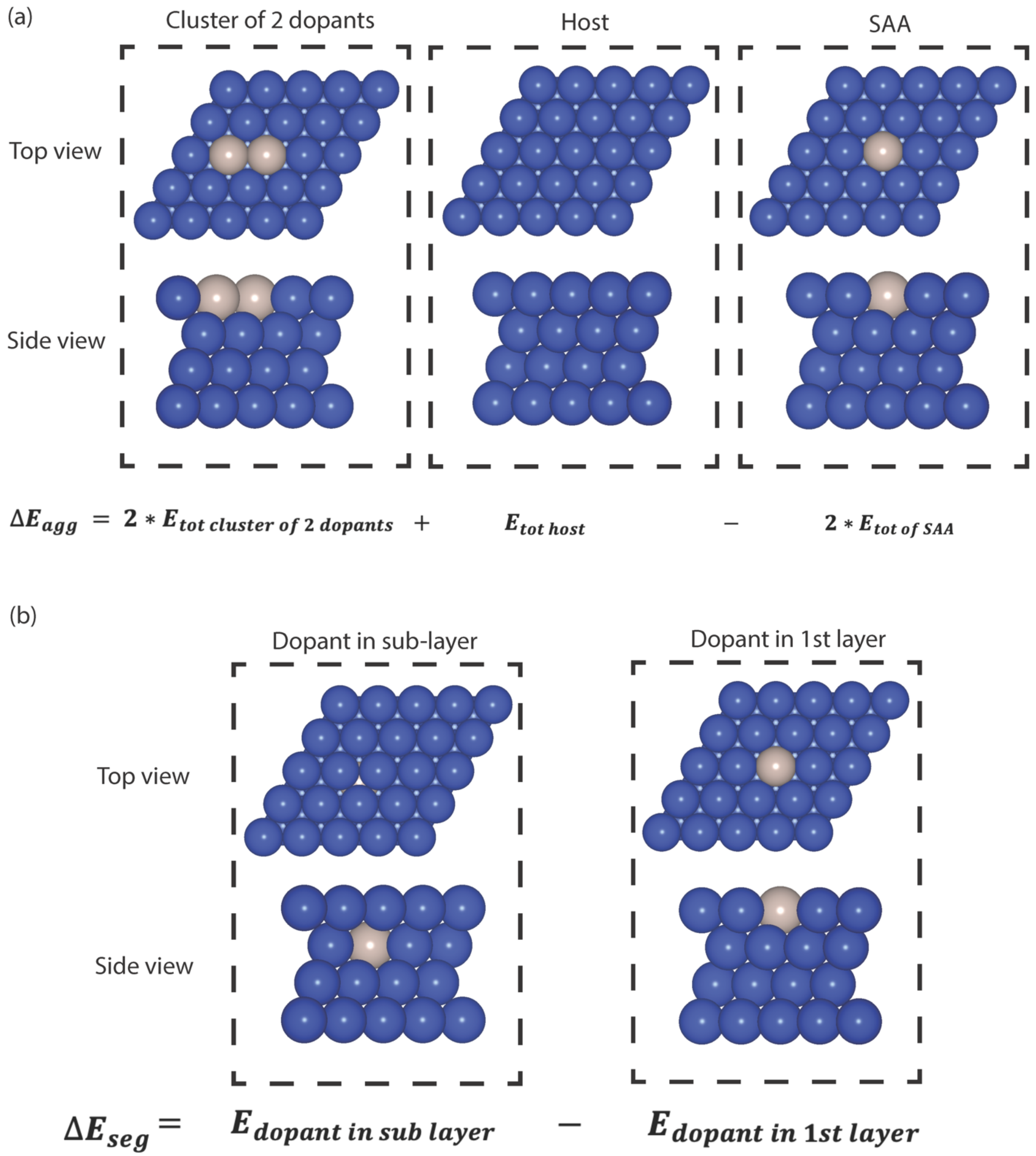

Figure S1. Representative slab models of top view (top) and side view (below) for calculating (a) aggregation energy and (b) segregation energy of single atom alloys (SAAs). Host atoms are colored as blue, and dopants are colored as gray.

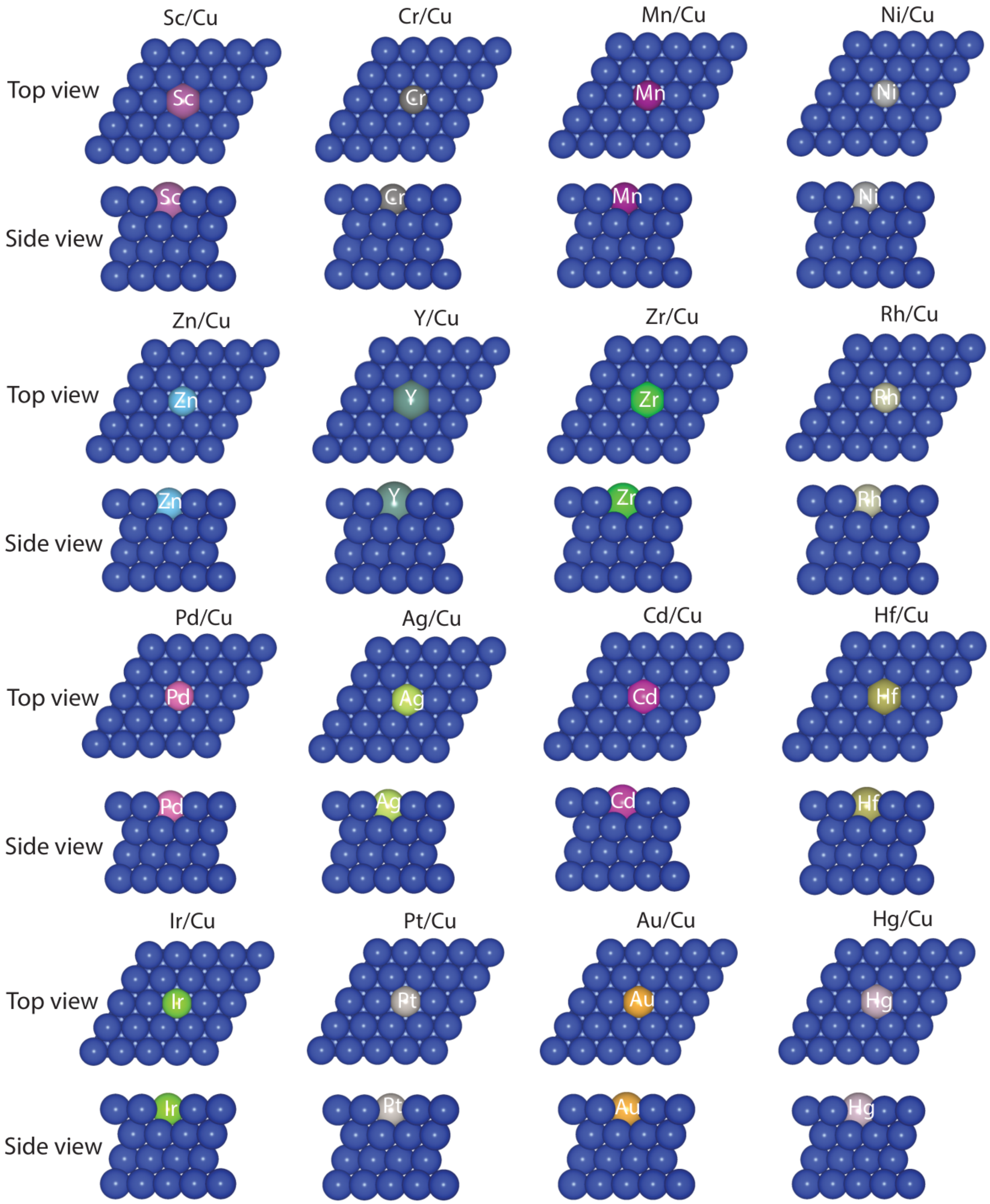

Figure S2. Geometries of top and side views of all stable SAAs formed by 3d, 4d, and 5d transition metals on Cu(111). Host Cu atoms are colored as blue, while dopants are shown in other colors.

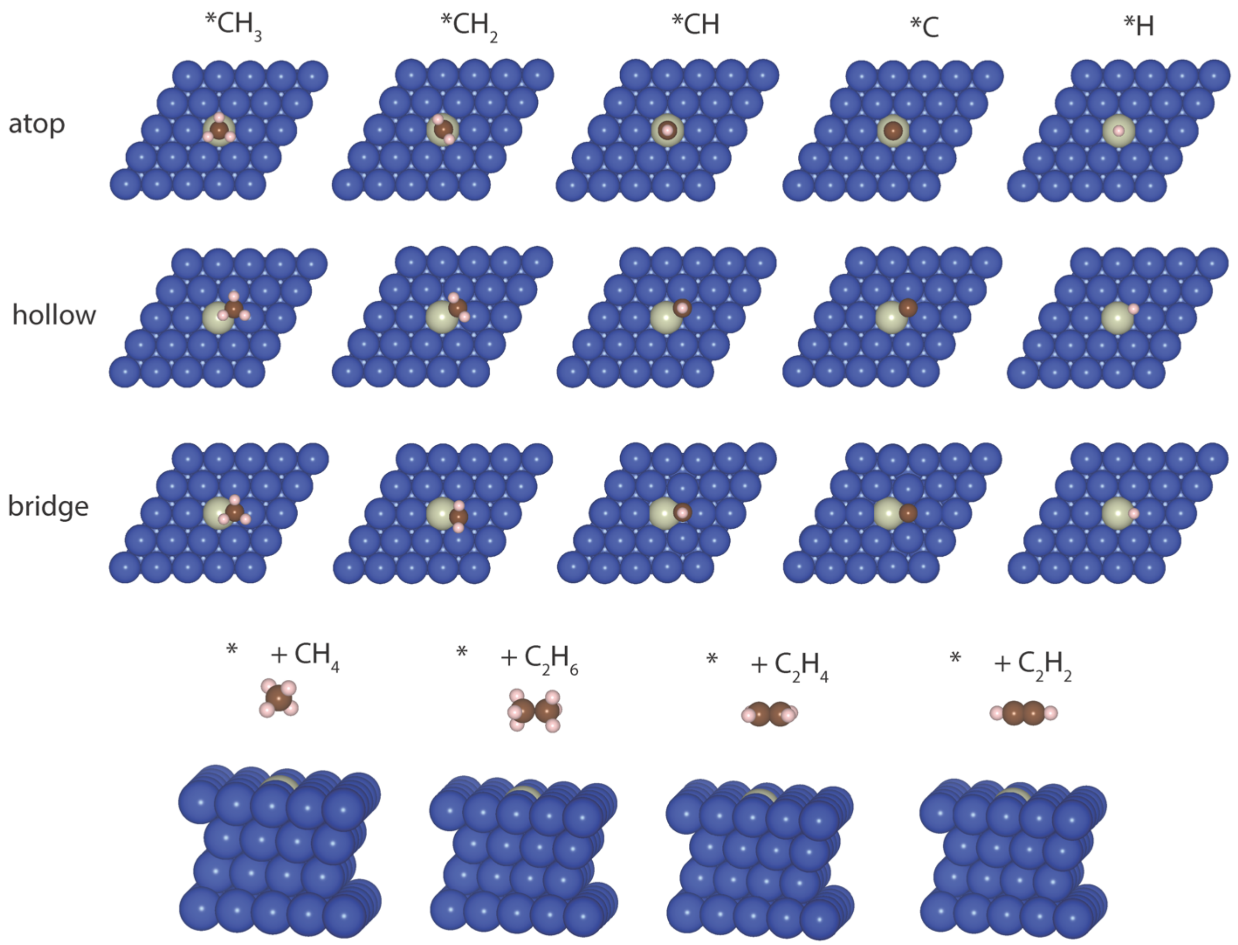

Figure S3. Representative geometries showing adsorption sites considered (i.e., atop, bridge, and hollow) for all CH_4 dehydrogenation intermediates, as well as desorbed molecules from the SAA surface. Atoms are colored as follows: blue for Cu, gray for dopant, brown for carbon, and light pink for hydrogen.

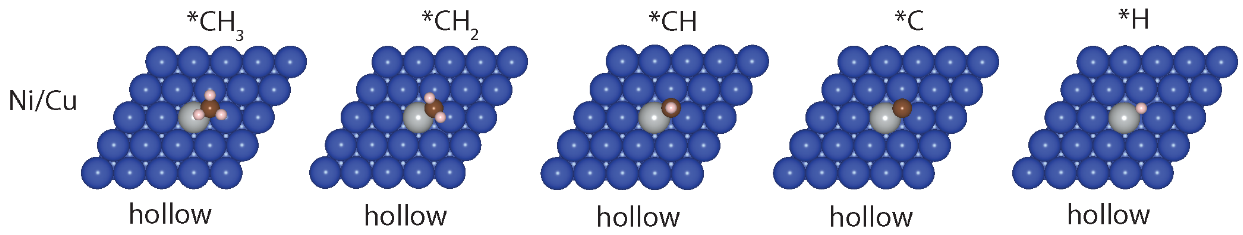

Figure S4. Geometries of favorable adsorption sites for all reaction intermediates on Ni/Cu SAA.

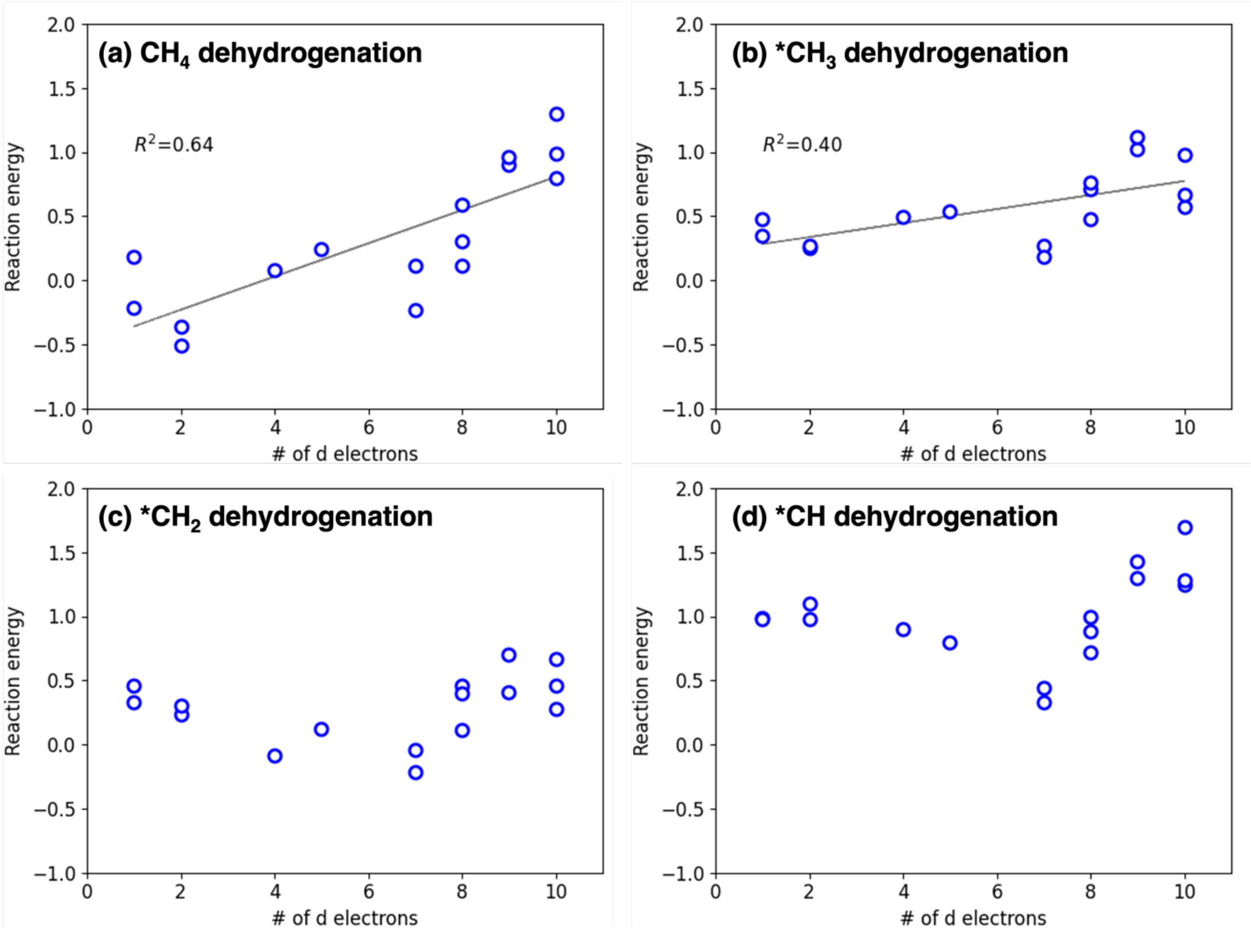

Figure S5. Correlation between reaction energies of (a) CH_4 dehydrogenation, (b) $*CH_3$ dehydrogenation, (c) $*CH_2$ dehydrogenation, (d) *CH dehydrogenation and number of dopant outer shell *d* electrons of SAAs. Gray lines indicate the best fit of a linear correlation with R^2 values shown on inset.

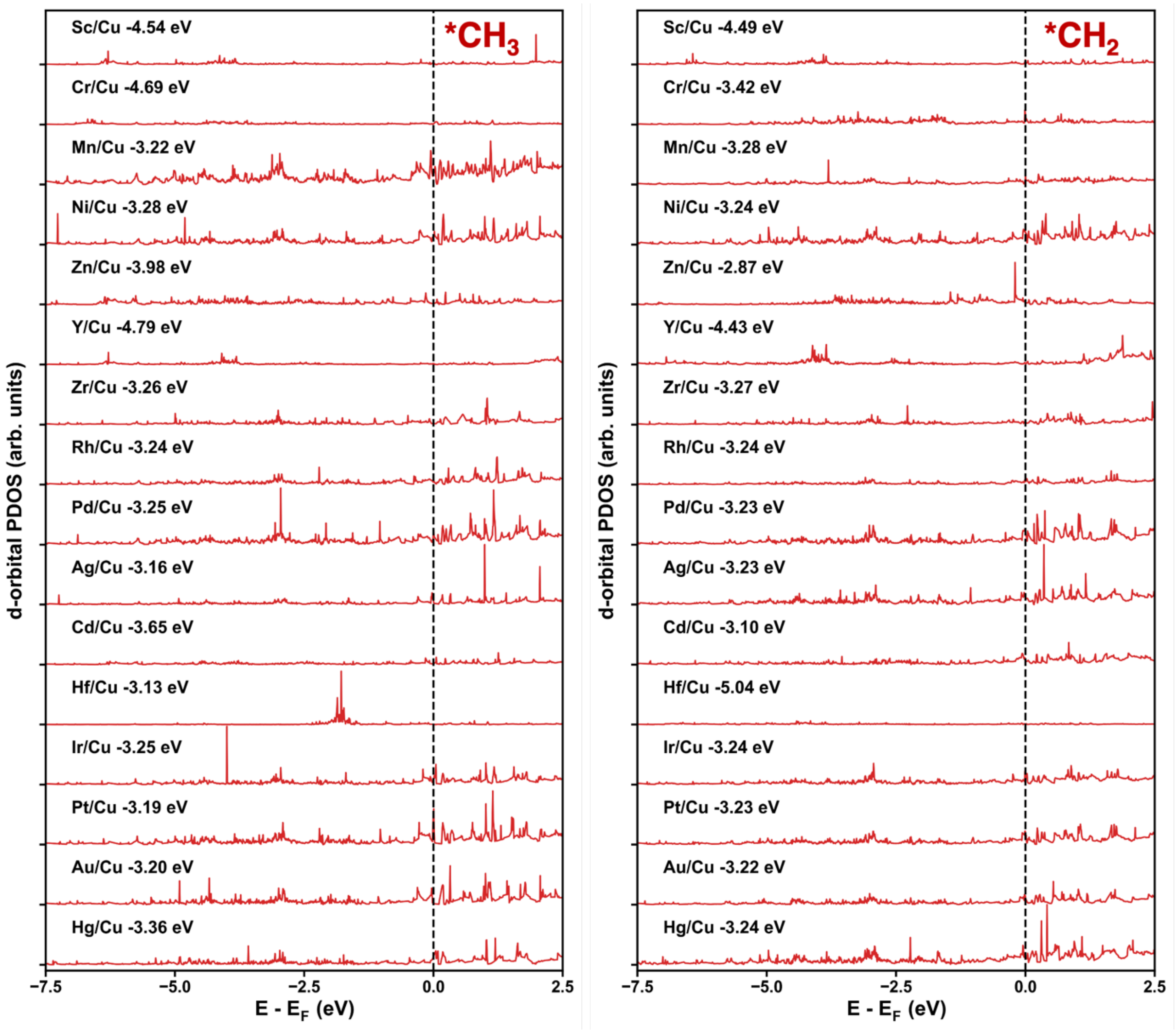
*CH3
*CH2
Sc/Cu -4.54 eV
Cr/Cu -4.69 eV
Mn/Cu -3.22 eV
Ni/Cu -3.28 eV
Zn/Cu -3.98 eV
Y/Cu -4.79 eV
Zr/Cu -3.26 eV
Rh/Cu -3.24 eV
Pd/Cu -3.25 eV
Ag/Cu -3.16 eV
Cd/Cu -3.65 eV
Hf/Cu -3.13 eV
Ir/Cu -3.25 eV
Pt/Cu -3.19 eV
Au/Cu -3.20 eV
Hg/Cu -3.36 eV
Sc/Cu -4.49 eV
Cr/Cu -3.42 eV
Mn/Cu -3.28 eV
Ni/Cu -3.24 eV
Zn/Cu -2.87 eV
Y/Cu -4.43 eV
Zr/Cu -3.27 eV
Rh/Cu -3.24 eV
Pd/Cu -3.23 eV
Ag/Cu -3.23 eV
Cd/Cu -3.10 eV
Hf/Cu -5.04 eV
Ir/Cu -3.24 eV
Pt/Cu -3.23 eV
Au/Cu -3.22 eV
Hg/Cu -3.24 eV
d-orbital PDOS (arb. units)
E - EF (eV)
−7.5
−5.0
−2.5
0.0
2.5

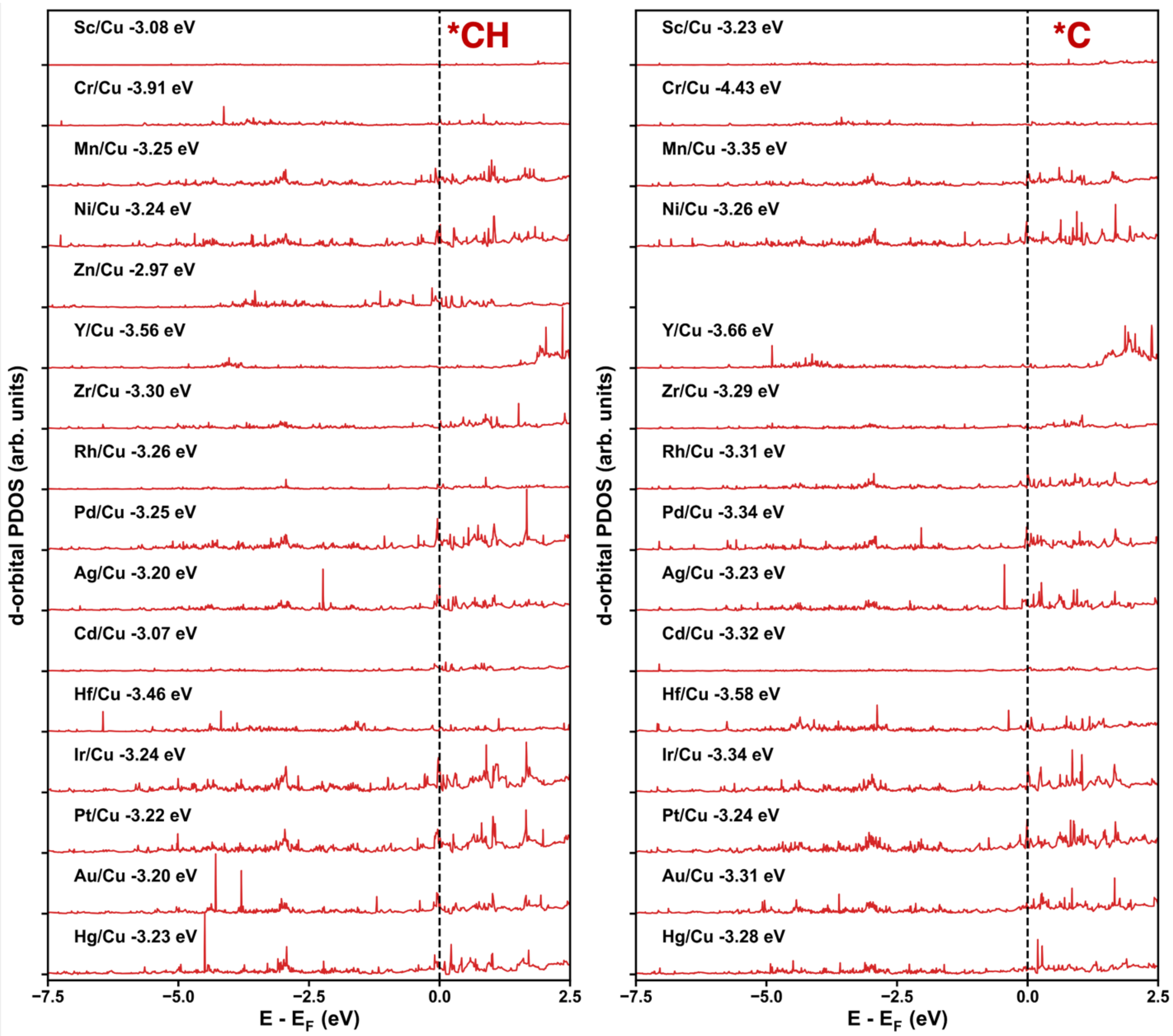

Figure S6. Projected density of states (PDOS) of dopant *d* orbitals of $*CH_3$-adsorbed SAA surface (top left), $*CH_2$-adsorbed SAA surface (top right), *CH-adsorbed SAA surface (bottom left), and *C-adsorbed SAA surface (bottom right). The *d*-band center is shown on inset.

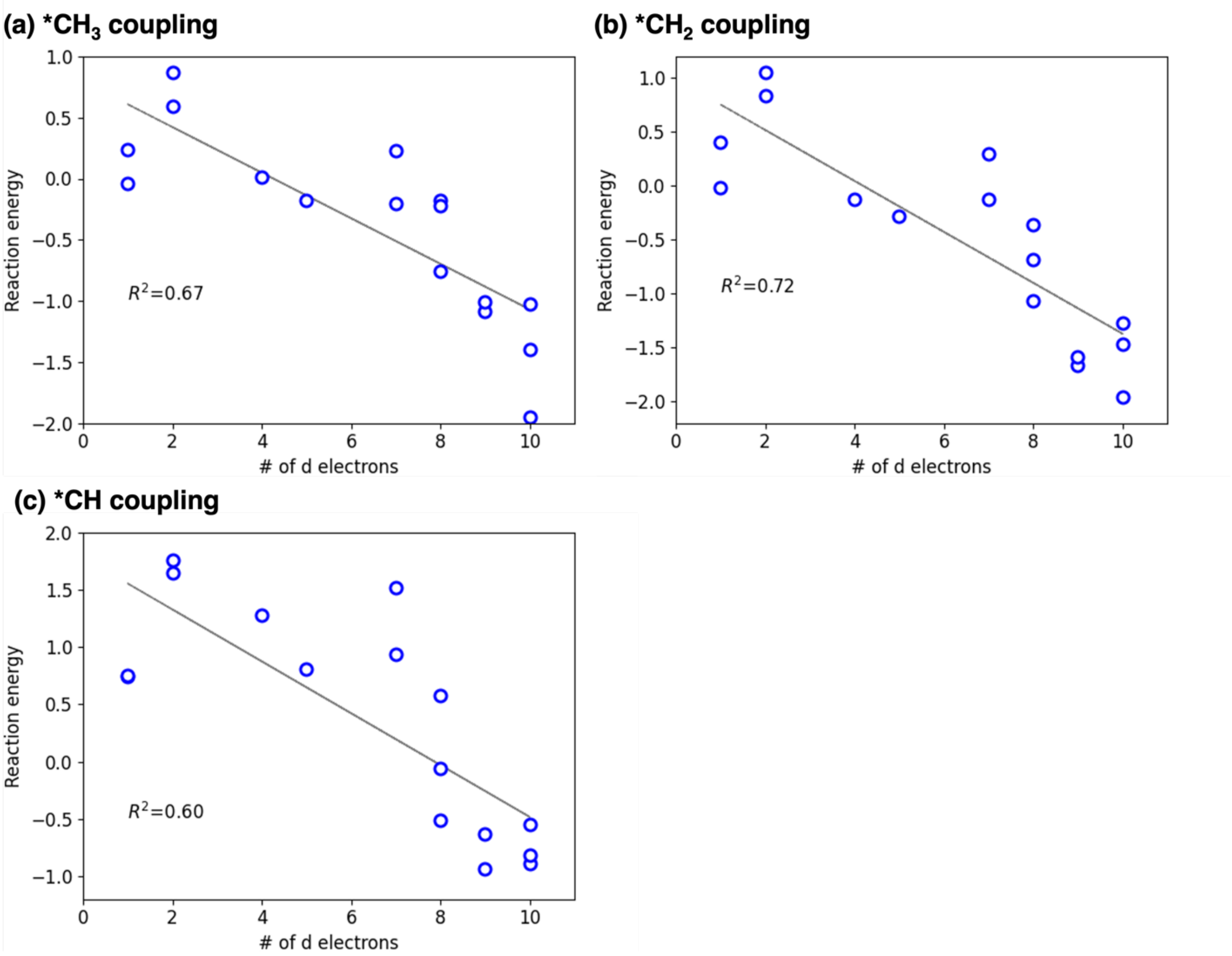

Figure S7. Correlation between reaction energies of (a) two $*CH_3$ coupling to ethane, (b) two $*CH_2$ coupling to ethylene, (c) two *CH coupling to acetylene and number of dopant outer shell *d* electrons of SAAs. Gray lines indicate the best fit of a linear correlation with R^2 values shown on inset.

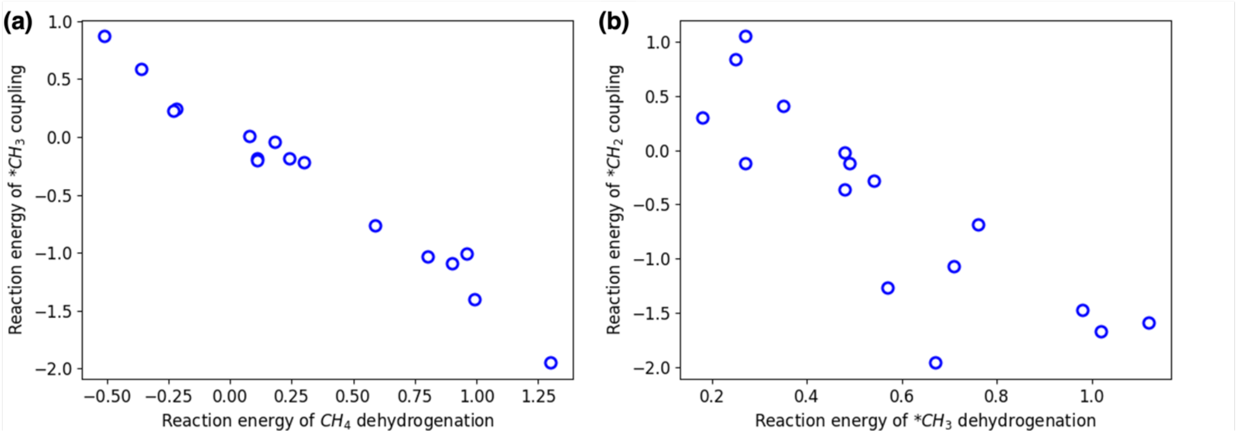

Figure S8. Correlation between reaction energies of (a) CH_4 dehydrogenation and *CH_3 coupling, and (b) *CH_3 dehydrogenation and *CH_2 coupling.

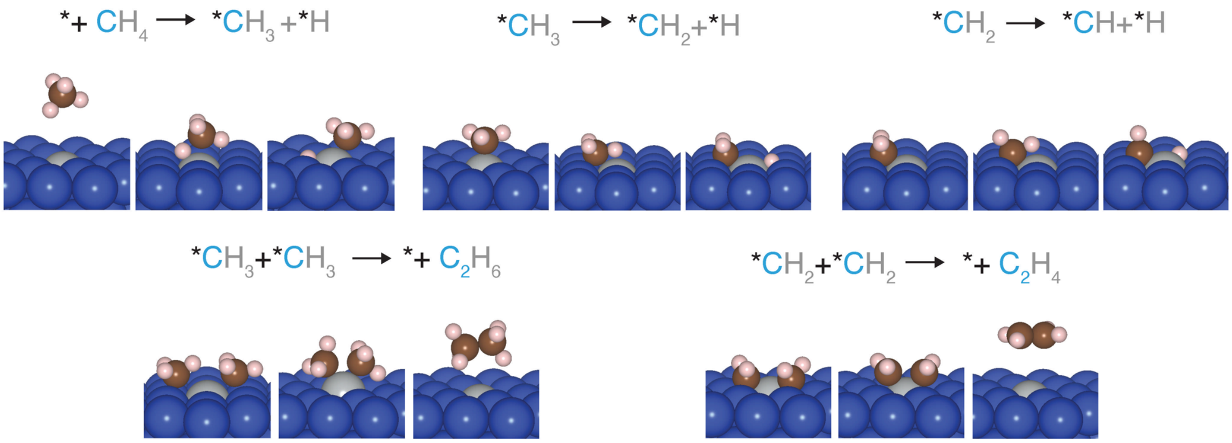

Figure S9. Critical structures of CH_4 dehydrogenation and C-C coupling mechanisms to C_2 products on Rh/Cu.

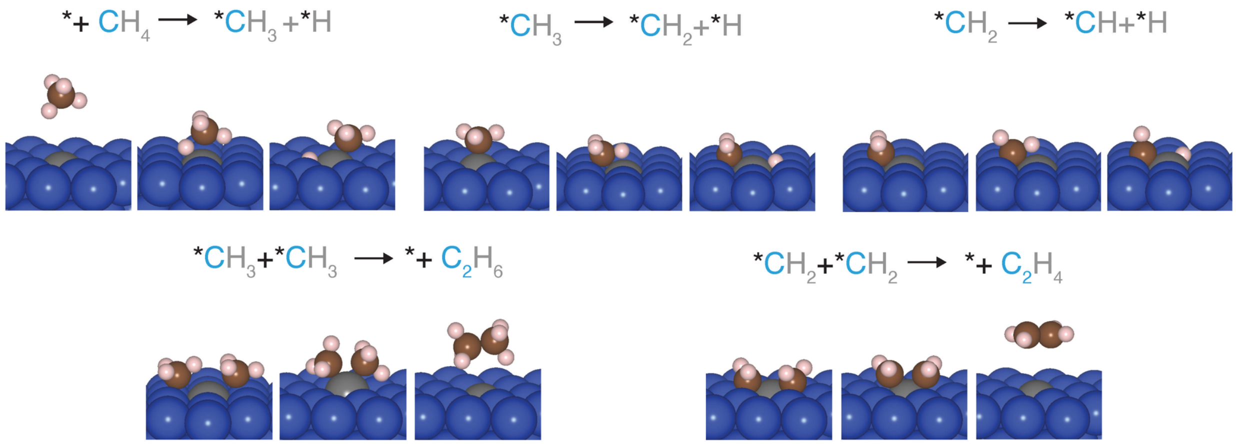

Figure S10. Critical structures of CH_4 dehydrogenation and C-C coupling mechanisms to C_2 products on Cr/Cu.

Table S1. DFT-PBE-D3-predicted aggregation and segregation energies of 27 SAAs with 3d-5d dopants on Cu(111) surface.

SAA	aggregation energy (eV)	segregation energy (eV)
Sc/Cu	0.71	0.33
Ti/Cu	0.02	-0.31
V/Cu	-0.72	-0.24
Cr/Cu	0.24	-0.05
Mn/Cu	0.17	0.033
Fe/Cu	-0.34	-0.12
Co/Cu	-0.62	-0.32
Ni/Cu	0.03	-0.19
Zn/Cu	0.07	0.25
Y/Cu	1.18	1.22
Zr/Cu	0.47	0.11
Nb/Cu	-0.80	-0.34
Mo/Cu	-2.12	-0.33
Ru/Cu	-0.59	-0.24
Rh/Cu	-0.05	-0.13
Pd/Cu	0.13	0.17
Ag/Cu	0.05	0.59
Cd/Cu	0.15	1.03
Hf/Cu	0.54	-0.10
Ta/Cu	-0.44	-0.48
W/Cu	-1.33	-0.44
Re/Cu	-1.37	-0.33
Os/Cu	-0.88	-0.26
Ir/Cu	-0.19	-0.12
Pt/Cu	0.16	0.18
Au/Cu	0.15	0.68
Hg/Cu	0.18	1.37

Table S2. The most favorable adsorption sites for all methane dehydrogenation intermediates on stable SAAs.

SAA	$*CH_3$	$*CH_2$	*CH	*C	*H
Sc/Cu	bridge	bridge	hollow	hollow	hollow
Cr/Cu	hollow	hollow	hollow	hollow	hollow
Mn/Cu	hollow	hollow	hollow	hollow	hollow
Ni/Cu	hollow	hollow	hollow	hollow	hollow
Zn/Cu	hollow	hollow	hollow	hollow	hollow
Y/Cu	bridge	bridge	hollow	hollow	hollow
Zr/Cu	atop	bridge	hollow	hollow	hollow
Rh/Cu	hollow	hollow	hollow	hollow	hollow
Pd/Cu	hollow	hollow	hollow	hollow	hollow
Ag/Cu	hollow	hollow	hollow	hollow	hollow
Cd/Cu	hollow	hollow	hollow	hollow	hollow
Hf/Cu	atop	bridge	hollow	hollow	hollow
Ir/Cu	atop	hollow	hollow	hollow	hollow
Pt/Cu	atop	hollow	hollow	hollow	hollow
Au/Cu	atop	hollow	hollow	hollow	hollow
Hg/Cu	atop	hollow	hollow	hollow	hollow

Table S3. Reaction energies in eV of all CH_4 dehydrogenation to *C and C-C coupling steps to ethane, ethylene, and acetylene.

SAA	CH_4 → $*CH_3$ + *H	$*CH_3$ → $*CH_2$ + *H	$*CH_2$ → *CH + *H	*CH → *C + *H	$*CH_3$ + CH_3 → C_2H_6	$*CH_2$ + $*CH_2$ → C_2H_4	*CH + *CH → C_2H_2
Sc/Cu	-0.02	0.35	0.46	0.99	0.24	0.41	0.74
Cr/Cu	0.08	0.49	-0.09	0.90	0.01	-0.12	1.28
Mn/Cu	0.24	0.54	0.12	0.80	-0.18	-0.28	0.81
Ni/Cu	0.11	0.48	0.11	0.72	-0.18	-0.36	0.58
Zn/Cu	0.80	0.98	0.67	1.70	-1.03	-1.47	-0.89
Y/Cu	0.18	0.48	0.33	0.98	-0.04	-0.02	0.75
Zr/Cu	-0.36	0.25	0.23	0.98	0.59	0.84	1.76
Rh/Cu	0.11	0.27	-0.04	0.44	-0.20	-0.12	0.94
Pd/Cu	0.59	0.71	0.46	1.00	-0.76	-1.07	-0.51
Ag/Cu	0.90	1.02	0.41	1.30	-1.09	-1.67	-0.63
Cd/Cu	0.99	0.57	0.46	1.25	-1.40	-1.27	-0.55
Hf/Cu	-0.51	0.27	0.30	1.10	0.87	1.05	1.65
Ir/Cu	-0.23	0.18	-0.22	0.33	0.23	0.30	1.52
Pt/Cu	0.30	0.76	0.40	0.88	-0.22	-0.68	-0.06
Au/Cu	0.96	1.12	0.70	1.43	-1.01	-1.59	-0.94
Hg/Cu	1.30	0.67	0.28	1.28	-1.95	-1.96	-0.82
